# Mechanistic insights into the kidney injury in chickens induced by hypervirulent fowl adenovirus serotype 4

**DOI:** 10.1128/spectrum.00058-25

**Published:** 2025-03-25

**Authors:** Chunhua Zhu, Jiayu Zhou, Zhen Chen, Cuiteng Chen, Ziyue Wang, Pei Yang, Guanghua Fu, Xiaodong Liu, Yu Huang, Chunhe Wan

**Affiliations:** 1 Fujian Provincial Key Laboratory for Avian Diseases Control and Prevention, Institute of Animal Husbandry and Veterinary Medicine, Fujian Academy of Agricultural Sciences, Fuzhou, China; 2 Institute of Biotechnology, Fujian Academy of Agricultural Sciences, Fuzhou, China; University of Georgia, Athens, Georgia, USA

**Keywords:** hypervirulent fowl adenovirus serotype 4, autophagosome-like vesicles' delivery, inflammation, kidney injury

## Abstract

Hypervirulent fowl adenovirus serotype 4 (FAdV-4) has emerged as a significant poultry pathogen since 2015, exhibiting clinical multi-organ and multi-tissue tropism post-infection, resulting in substantial economic losses in the poultry industry. However, the molecular mechanism underlying kidney injury caused by FAdV-4 infection remains unclear. Our results indicated that FAdV-4 infection in chickens induces damage to kidney tissues, characterized by the degeneration and necrosis of kidney epithelial cells, glomerular injury, endoplasmic reticulum stress, and the activation of a robust inflammatory response in the kidney cells. Notably, autophagosome-like vesicles enclosed clusters of viral particles that were transmitted between kidney cells post-infection. There might be a novel mechanism of vesicle-mediated cell-to-cell transmission of hypervirulent FAdV-4 that hijacks autophagosome-like vesicles. We also investigated cellular autophagy in kidney cells *in vivo* and *in vitro* during early FAdV-4 infection. The autophagy-related marker proteins LC3B, ATG5, and BECN1 were upregulated post-infection, whereas SQSTM1 was downregulated, indicating that FAdV-4 infection enhances autophagic flux and induces complete autophagy. The viral structural protein Fiber 2 was also observed to colocalize with the autophagy-related marker protein LC3B and the exosome-specific marker protein CD63 in the kidney cells at 24 hpi, suggesting that FAdV-4-induced cellular autophagy promotes viral replication in kidney cells and that autophagosome-like vesicles are involved in early FAdV-4 replication *in vivo* in chickens. Our results offer novel insights into the pathogenesis of hypervirulent FAdV-4 from the perspective of kidney injury post-infection.

**IMPORTANCE:**

Hypervirulent fowl adenovirus serotype 4 (FAdV-4) has become globally prevalent since 2015 as a predominant pathogen on poultry farms, leading to substantial economic losses for the poultry industry. However, the molecular mechanisms underlying kidney injury induced by FAdV-4 infection remain unclear. In this study, we primarily elucidated the mechanisms of kidney injury induced by FAdV-4 infection in chickens, utilizing both *in vitro* and *in vivo* models. Our results demonstrate that FAdV-4 infection in chickens causes degeneration and necrosis of kidney epithelial cells, glomerular injury, and expansion of the endoplasmic reticulum, while also triggering a robust inflammatory response in kidney cells. Notably, we observed the cell-to-cell transmission of virus particles delivered by autophagosome-like vesicles, and the viral infection-induced cellular autophagy facilitated viral replication in the kidney cells. These findings offer a novel perspective to understand the molecular mechanisms of FAdV-4-induced kidney injury and establish a basis for further investigation into the molecular pathogenesis of hypervirulent FAdV-4.

## INTRODUCTION

Hypervirulent fowl adenovirus serotype 4 (FAdV-4) was first reported in Ankara, Pakistan, in 1987 and is also known as “Ankara disease” (1). FAdV-4 has become globally prevalent since 2015, with documented infections in Japan (2, 3), China (4, 5), the United States (6), South Korea (7), Canada (8), and other nations. It is a nonenveloped, dicosahedral, and double-stranded DNA virus belonging to the genus *Aviadenovirus*, which commonly infects chickens, ducks, geese, and peacocks. The FAdV-4 is categorized into two types based on its virulence: pathogenic and nonpathogenic strains. Since 2015, a novel genotype of virulent FAdV-4 with hydropericardium syndrome has emerged in China and has become a predominant pathogen on poultry farms (9). This novel virulent genotype strain spreads quickly, with a wide range of multi-organ and multi-tissue tropism. It replicates in the liver, kidney, spleen, lung, and other tissues, leading to the production of high viral titers. These infections are associated with pathologies such as hepatitis-hydropericardium syndrome and nephritis, with mortality rates ranging from 30% to 100% (10, 11), resulting in substantial economic losses for the poultry industry.

Recent studies of FAdV-4 have focused on pathogenicity (12, 13), innate immunity (14), and vaccines (15). Despite ongoing efforts to contain the occurrence and transmission of FAdV-4 disease, further elucidation of the molecular pathogenesis of FAdV-4 is still crucial for disease prevention and control. Previous research has demonstrated the liver injury caused by FAdV-4 infection, revealing that the activation of the LXR-α signaling pathway post-infection leads to the accumulation of triglyceride in liver cells and subsequent liver steatosis (12, 16). These findings suggest that a robust inflammatory response and lipid accumulation in the liver play significant roles in the pathogenesis of FAdV-4. However, the molecular mechanism underlying kidney injury induced by FAdV-4 infection remains unclear.

Autophagy is a highly conserved catabolic degradation process in which bilayer membrane vesicles are generated that coat and isolate pathogens, abnormal proteins, and organelles for degradation in lysosomes, thus maintaining cellular homeostasis. Autophagy is induced by various stress stimuli, including endoplasmic reticulum (ER) stress, starvation, oxidative stress, and viral infections (17). Autophagy has been a double-edged sword in viral evolution, and various strategies have been developed to either enhance or restrict viral replication (18, 19). For instance, the egg drop syndrome virus activates autophagy in duck embryo fibroblasts through the PI3K/AKT/MTOR pathway to facilitate viral replication (20). Several studies have also shown the significant role of autophagy in FAdV-4 infection (21, 22), in which the viral hexon protein interacts with host BAG3 to induce cellular autophagy and promote viral replication in LMH cells (23). In contrast, the binding of the viral protein VP2 to the Avibirnavirus receptor triggers autophagy, leading to the inhibition of viral replication (24). Despite these findings, the precise molecular mechanism by which hypervirulent FAdV-4 induces cellular autophagy remains unclear.

Autophagy not only triggers the innate immune response but also breaks down cellular components to create an environment suitable for eliminating invaders (25). However, the excessive activation or inhibition of autophagy can result in ER stress and mitochondrial dysfunction, which is detrimental to the host, leading to tissue damage and potential death. Research has shown a correlation between autophagy and kidney injury (26, 27). The role of cellular autophagy in the pathogenesis of various poultry diseases, including kidney injury, is an area of significant interest (26). However, it remains unclear how autophagy influences different patterns of cell death in kidney injury and what molecular interactions occur during dynamic homeostasis to affect overall cell fate. Several viruses have evolved mechanisms to either prevent the formation of autophagosomes or to hijack them for their own survival and replication, even utilizing the metabolites and energy generated by autophagy for their benefit. Different viruses manipulate autophagy to adjust host cellular life cycles and pathogenesis, although the precise molecular mechanisms remain unclear. The interrelation between autophagy and infection is more intricate than previously thought, and viruses have evolved to effectively subvert and disrupt autophagy (19). Studies have shown that interferon gamma (IFN-γ)-induced GTPases inhibit viral replication by targeting the *Norovirus* replication complex with LC3 (28). This research has shed light on how autophagy (specifically, the LC3-coupled system) and the innate immune response (IFN-induced GTPases) collaborate to combat viral infections.

In this study, we primarily elucidated the underlying molecular mechanism of the kidney injury induced by FAdV-4 infection in specific-pathogen-free (SPF) chickens, using both *in vitro* and *in vivo* models. Our results showed that FAdV-4 infection of chickens causes the degeneration and necrosis of kidney epithelial cells, glomerular injury, expansion of the ER, and a robust inflammatory response in kidney cells. Notably, we observed the cell-to-cell transmission of virus particles mediated by autophagosome-like vesicles, which facilitated the movement of viral particles among primary chick kidney cells, thus increasing the efficiency of hypervirulent FAdV-4 infection. The viral infection-induced cellular autophagy facilitated viral replication in the kidney cells. These findings offer a novel perspective to understand the molecular mechanisms of FAdV-4-induced kidney injury and establish a basis for further investigation into the molecular pathogenesis of hypervirulent FAdV-4.

## MATERIALS AND METHODS

### Strains and animals

The FAdV-4 strain (GenBank ID: MG856954.1) was isolated from Meizhou City, Guangdong Province, and maintained by the Fujian Key Laboratory for Avian Diseases Control and Prevention, Fujian Academy of Agricultural Sciences (11). SPF chickens and SPF chick embryos were purchased from Jinan SPAFAS Poultry Co., Ltd (Shandong, China).

### Ultrastructural analysis of primary chick kidney cells post-infection

Kidneys were aseptically removed with tweezers from two 18-day-old SPF chick embryos. The extracted kidney tissues were rinsed with Dulbecco’s modified Eagle’s medium (DMEM) three times before they were transferred to a new Petri dish and cut into small pieces. Trypsin (0.25%, 2 mL) was added to digest the kidney tissues for 2 min for five cycles. The cell suspension was centrifuged at 1,200 × *g* for 3 min to obtain the cell precipitate. A volume of 5 mL of DMEM containing 10% fetal bovine serum and 1% penicillin/streptomycin was added to disperse the cells, and the cell density was adjusted to 1 × 10^6^/mL and cultured in a CO_2_ incubator. After the primary chick kidney cells had been cultured for approximately 24 h and had formed a fresh monolayer, they were inoculated with two median tissue culture infective doses (2 TCID_50_) of FAdV-4 viral suspension. The cytopathogenic changes in the primary chick kidney cells were observed under a microscope at 48 hpi. To investigate the replication dynamic changes of the FAdV-4 in primary chick kidney cells, the viral suspensions were collected at the indicated times (12, 24, 48, 72, 96, and 120 h) post-infection. The viral loads of FAdV-4 in collected suspensions were determined by the qPCR method (11).

After inoculation with FAdV-4, both the infected cells and normal control cells were washed twice with phosphate-buffered saline (PBS), digested with 0.25% trypsin for 150 s, and then centrifuged at 1,200 × *g* at room temperature for 3 min to obtain the cell precipitates. Ultrathin sections of the cells were prepared after 500 µL of electron microscopy fixing solution containing 5% glutaraldehyde was added. The sections were washed three times with PBS, fixed with 1% osmic acid for 1.5 h, washed again three times with ddH_2_O, and dehydrated with a graded series of alcohol. The sections were then stained with 3% uranyl acetate and lead citrate and washed for 5 min with ddH_2_O. The ultrastructure of the primary chick kidney cells after FAdV-4 infection was observed under an electron microscope (Hitachi HT7700, Tokyo, Japan) at 80 kV. Autophagosome-like vesicles are defined as double-membraned or single-membraned vesicles with a diameter of 0.3–2.0 µm.

### Histopathological analysis of the kidney post-infection

Thirty-five-day-old SPF chickens were divided into two groups for histopathological analysis. One group was injected intramuscularly with 0.5 × 10^3.8^ TCID_50_/mL FAdV-4 suspension in an injection dose of 0.2 mL per chicken. The control group was injected with 0.2 mL of PBS. Kidney tissue samples were collected 24 h after the viral challenge, fixed in 4% paraformaldehyde (Sangon Biotech, Shanghai, China) at 4°C for 24 h, and then processed into paraffin sections for hematoxylin–eosin (HE) staining to observe the early pathological injury in kidney tissue after FAdV-4 infection. The detailed HE staining method used was based on reference (11).

### Immunohistochemical assay

Paraffin sections were prepared from infected chicken kidney tissues and normal kidney tissues 24 h after the viral challenge. Antigen repair was conducted after dewaxing, and the sections were treated with 4% bovine serum albumin (BSA) for 45 min. A rabbit anti-fiber-2 polyclonal antibody (diluted 1:500; primary antibody) was then added, and the samples were incubated overnight at 4°C. A goat anti-rabbit horseradish peroxidase (HRP)-conjugated secondary antibody (Sigma-Aldrich; diluted 1:10,000) was then added, and the samples were incubated at 37°C for 45 min. After the sections were washed four times with PBS, they were stained with 3,3′-diaminobenzidine and then with hematoxylin for 10 min. After decolorization, the tissue sections were dehydrated in a gradient of alcohol, rendered transparent with xylene, and sealed with neutral gum. The histopathology before and after FAdV-4 infection was compared microscopically, with a brown-yellow color indicating a positive signal of Fiber2 protein post-FAdV-4 infection.

### Immunofluorescence assay

To investigate the relationship between autophagy and viral replication in kidney tissue after FAdV-4 infection, colocalization of the viral Fiber2 protein with the host autophagy-related proteins LC3B (Abcam, Shanghai, China) and CD63 (Bioss, Beijing, China) was examined with an immunofluorescence assay. The kidney tissues from SPF chickens infected with FAdV-4 for 24 h were collected, cut into 3 × 3 × 3 mm^3^ sections, fixed in paraformaldehyde (Sangon Biotech, Shanghai, China) at 4°C for 24 h, and processed into paraffin sections. After antigen retrieval, the sections were coated with 2% BSA for 120 min. A rabbit anti-Fiber2 antibody of FAdV-4 was added and incubated for 60 min, followed by a rabbit anti-LC3B polyclonal antibody (ab63817, Santa Cruz, USA) and a rabbit anti-CD63 polyclonal antibody (bs-23032R, Beijing, China) for 60 min each. Goat anti-rabbit secondary antibodies conjugated with Alexa Fluor 488 or cyanine 3 (Cy3) were added, and the samples were incubated for 60 min. The cell nuclei were counterstained with 4′,6-diamidino-2-phenylindole (DAPI) for 10 min. The sections were then sealed and examined with confocal immunofluorescence microscopy (TCS SP8; Leica Microsystems GmbH, Wetzlar, Germany) to observe the positive fluorescent signals.

### Quantitative real-time PCR

Quantitative real-time PCR was used to assess the expression levels of inflammatory factors (IL-1β, IL-2, IL-6, IL-8, and TNF-α), as well as antiviral cytokines (IFN-α, IFN-γ, MX-1, and OASL), to investigate the innate immune response in the kidneys of FAdV-4-infected chickens. A total of 50 mg of kidney tissue was obtained from chickens (*n* = 3 birds for each group) at various time points (6, 12, and 24 hpi). Each kidney tissue sample was immersed in 500 µL of Trizol (Invitrogen, Carlsbad, USA) and quickly frozen in liquid nitrogen. Total RNA was then extracted from every sample and reverse transcribed into cDNA using a reagent kit (Takara, Dalian, China). The RT-qPCR reaction protocols and primers applied in this investigation were based on reference (11). The relative mRNA expression levels of the inflammatory factors (IL-1β, IL-2, IL-6, and IL-8) and antiviral cytokines (TNF-α, IFN-α, IFN-γ, MX-1, and OASL) were calculated utilizing the 2^-ΔΔCt^ method. Data analysis was performed using two-way ANOVA with Prism 8 software (GraphPad Software, Inc., San Diego, CA, USA).

### Western blotting analysis

The expression levels of autophagy-related factors LC3B, SQSTM1, BECN1, ATG5, and GRP78 in the kidneys of SPF chickens infected with FAdV-4 were analyzed with western blotting. Kidney tissue samples (100 mg) were homogenized, and RIPA lysate containing 1 mM PMSF (Servicebio, Wuhan, China) was added for cell lysis for 10 min on ice. After centrifugation at 12,000 × *g* at 4°C for 10 min, the supernatants were collected, and the protein concentrations were adjusted to 2 mg/mL, with a loading sample volume of 10 µL per well. Electrophoresis was performed on an SDS-PAGE gel, and the proteins were transferred at 200 mA to polyvinylidene difluoride (PVDF) membranes (Millipore, Bedford, USA) for 12 h. The membranes were then blocked with 5% skim milk solution (Servicebio, Wuhan, China) for 2 h. Primary antibodies (directed against LC3B [Abcam]; SQSTM1, Beclin1, ATG5, GRP78 [Bioss]; and Fiber2) were diluted 1:1,000, added to the PVDF membranes, and incubated overnight at 4°C. After the membranes were washed three times with PBS containing Tween-20, HRP-conjugated secondary antibodies (Sigma-Aldrich; diluted 1:10,000) were added, and the membranes were incubated with shaking for 2 h. The membranes were washed three times with Tris-buffered saline containing Tween 20, and an enhanced chemiluminescence kit (Beyotime Biotechnology, Beijing, China) was used to visualize the color reaction. The protein bands were captured with a gel imaging system and quantified with the ImageJ software.

### Impact of cellular autophagy regulators on FAdV-4 replication

To determine whether the autophagy regulators rapamycin and 3-MA affect the activities of primary kidney cells and whether the pharmacological alteration of autophagy affects cell viability, we used a Cell Counting Kit-8 (CCK-8) kit (Sangon Biotech, Shanghai, China) according to the manufacturer’s instructions. In brief, primary chick kidney cells were diluted to 1 × 10^5^ cells/mL. After the primary cells had grown into a fresh monolayer, they were treated with gradient concentrations of rapamycin (5, 10, 25, and 50 nM) or 3-MA (1, 2, 3, and 4 mM) for 48 h. The viability of the primary chick kidney cells was determined with the CCK-8 assay to determine the suitable concentrations of autophagy regulators. Five biological replicates were prepared for each concentration, with 100 µL of cell suspension per well. After 48 hpi, the plates were washed with PBS to remove the autophagy regulators, and 100 µL of DMEM containing 10% fetal bovine serum and 1% penicillin/streptomycin was added. The CCK-8 solution (10 µL) was then added to each well (Sangon Biotech, Shanghai, China). After gentle mixing, the plates were further incubated at 37°C in a 5% CO_2_ incubator (Thermo Fisher Scientific, USA) for 4 h. The optical density at a wavelength of 450 nm (OD_450_) of each well was then measured with a microplate reader (Infinite 200 Pro, servoLAB GmbH, Kumberg, Austria).

After the primary chick kidney cells had grown to fresh monolayers in a 6-well plate, optimized solutions of the autophagy regulators rapamycin and 3-MA were added to the cultured cells. The cells were then cultured in a 5% CO_2_ incubator for 4 h, treated with 2 TCID_50_ FAdV-4, and cultured at 37°C for 2 h. The supernatant was removed and replaced with DMEM containing 2% fetal bovine serum for maintenance culture. Samples were collected at 24 and 48 h, respectively. The protein expression levels of LC3B, SQSTM1, Beclin1, ATG5, and GRP78 were assessed with western blotting. The expression of fiber 2 before and after treatment with the autophagy regulators was also measured.

### Statistical analysis

The data were analyzed with the two-way analysis of variance (ANOVA) using GraphPad Prism 8.0.2 software. The data are presented as the means ± standard deviation of three independent experiments. ns, *P* > 0.05; *P < 0.05; **P < 0.01; and ***P < 0.001.

## RESULTS

### Ultrastructural analysis of kidney cells infected by FAdV-4

**Fig 1:**
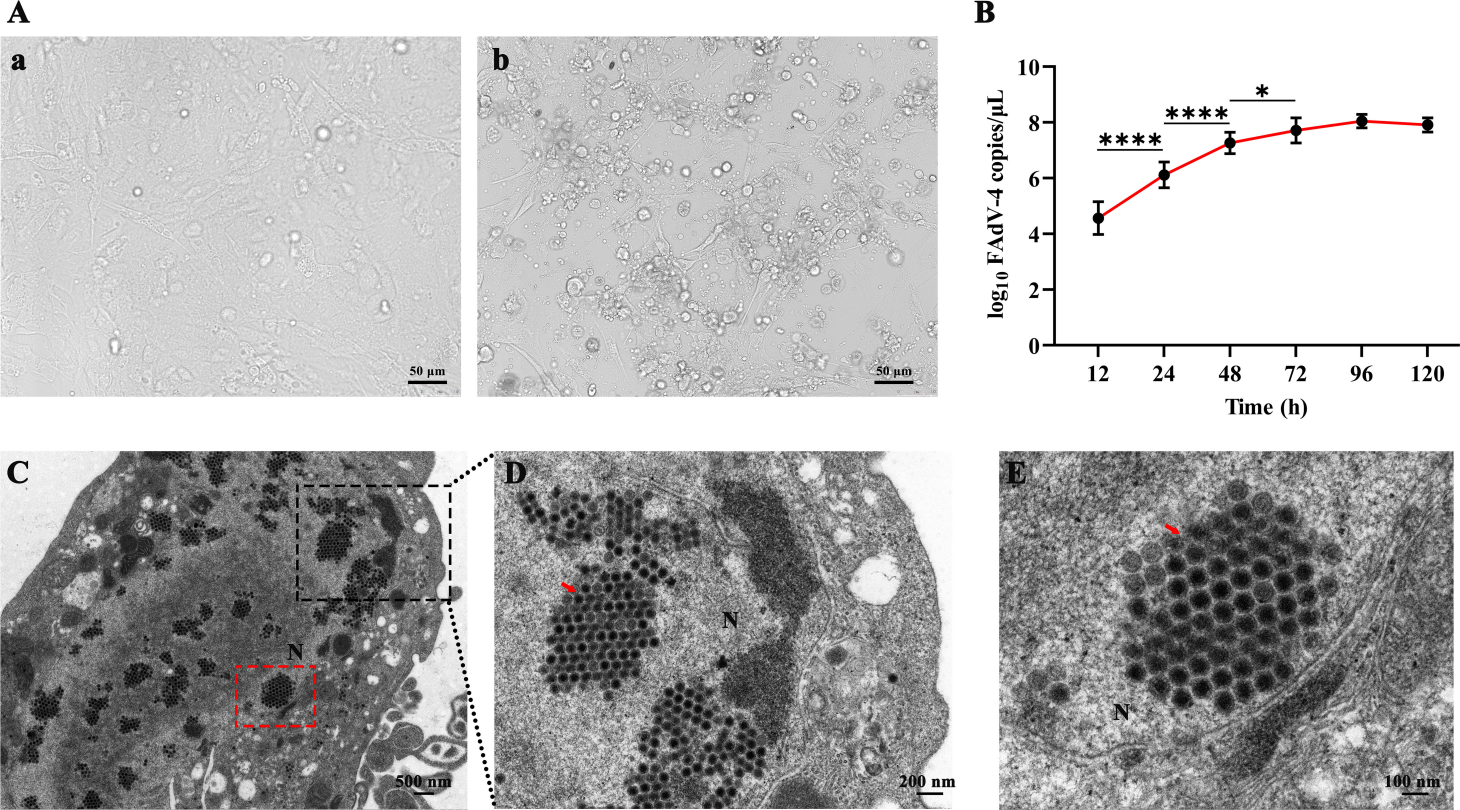
Virus culture and electron microscope observation. (A) The typical cytopathic effects post-FAdV-4 infection. Scale bar, 50 µm. (B) The growth curve of FAdV-4-infected primary kidney cells at the indicated times using qPCR. Data are means ± SD of the results of three independent experiments. (C) The virus particles are arranged in the form of a crystal lattice pattern in the nucleus. (D and E) Local magnification views of panel C; black and red dotted rectangles represent panels D and E, respectively.

Typical cytopathic effects were observed at 48 hpi, the primary chick kidney cells were enlarged and formed clumps, characteristic of cytopathic lesions (Fig. 1A). The viral loads significantly increased at 24 and 48 hpi, suggesting that the FAdV-4 were in the logarithmic growth phase (Fig. 1B). Assemblies of virus particles were observed in the nucleus at 48 hpi, with a clearly visible virus assembly factory in the single-cell ultrastructure (Fig. 1C through E). These virus particles were approximately 70 nm in size and formed crystalline arrays. Following infection with FAdV-4, the nuclear membrane ruptured and gradually disintegrated as compared to normal cells. The ER exhibited expansion and abnormality post-infection. The infected cells had numerous vacuoles and discontinuous nuclear membranes in the cytoplasm after virus infection, with progeny viruses being released from the damaged nucleus through the cell membrane.

**Fig 2:**
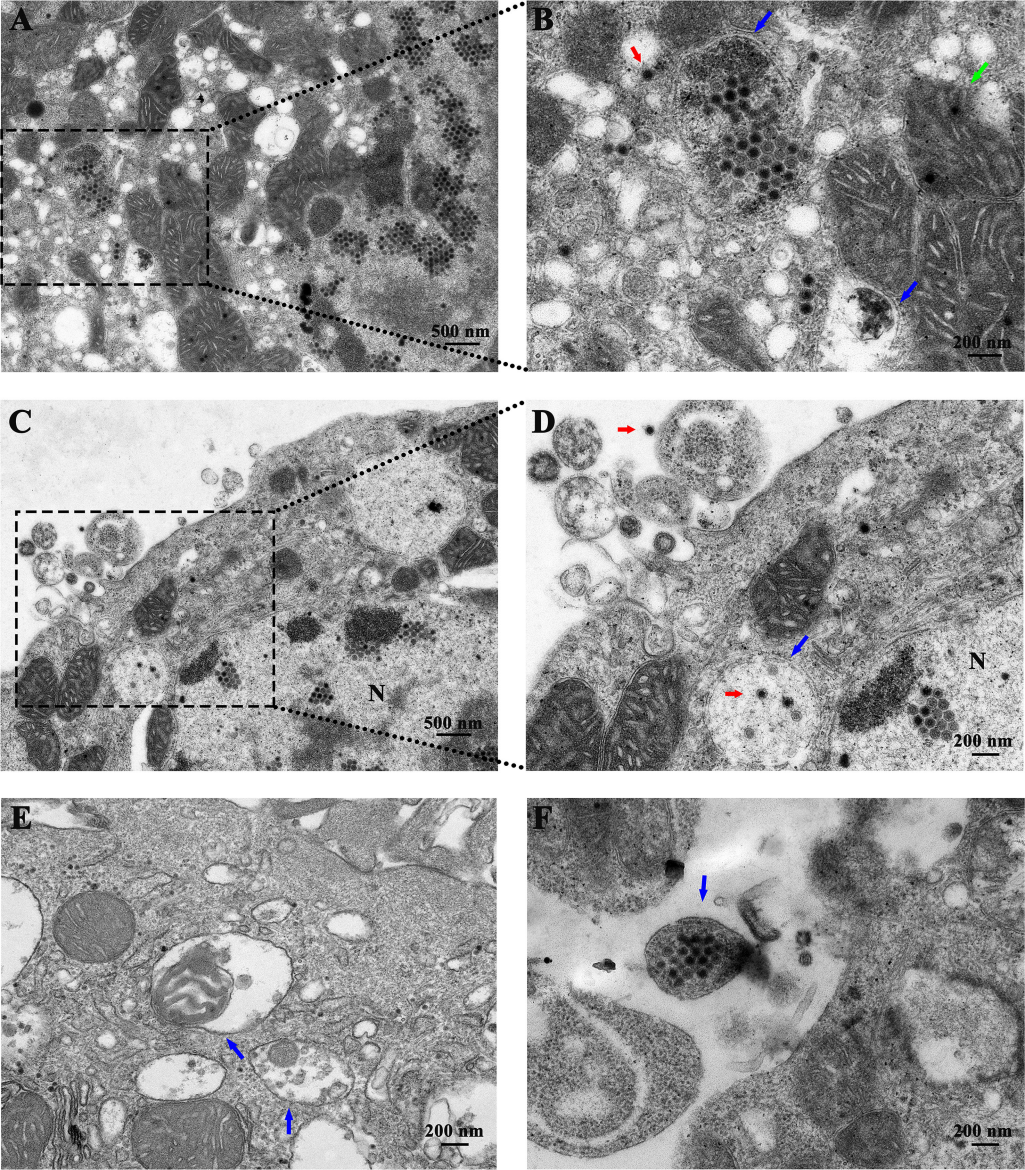
Ultrastructural analysis in primary chick kidney infected with FAdV-4. (A) Autophagosome-like structures in the cytoplasm. (B) Autophagosome-like structures are wrapping the virus particles. Local magnification views of panel A. Mitochondria swell was observed post-FAdV-4 infection. (C) Vesicles that transport viral particles in the cytoplasm in the primary chick kidney cells post-infection. (D) Enlargement of autophagosome-like structure of panel C (black dotted rectangles). (E) Autophagosome-like vesicles contain damaged mitochondria in the cytoplasm. (F) Extracellular vesicle facilitated FAdV-4 transmission cell to cell. N, nucleus. Blue arrow, autophagosome-like vesicles contain viral particles or damaged organelles. Red arrows, virus particles. Green arrows, mitochondria infected by FAdV-4.

Additionally, mitochondria in the cytoplasm were deformed and swollen, with disordered ridge structures, and some mitochondria even disappeared or formed vacuoles (Fig. 2). Notably, virus particles were observed within some of the mitochondria, suggesting that viral infection may cause mitochondrial injury.

Autophagosome-like vesicles were observed in the cytoplasm following viral infection and appeared to contain damaged organelles, cell fragments, or viral particles, indicating that cellular autophagy was activated post-infection (Fig. 2, blue arrow). FAdV-4 infection triggered autophagic flux. Notably, these vesicles were observed in both the kidney cytoplasm and extracellular space and delivered a significant number of virus particles, suggesting a potential mechanism for the infection and transmission of the hypervirulent FAdV-4.

### Histopathological analysis of the kidney post-infection

**Fig 3:**
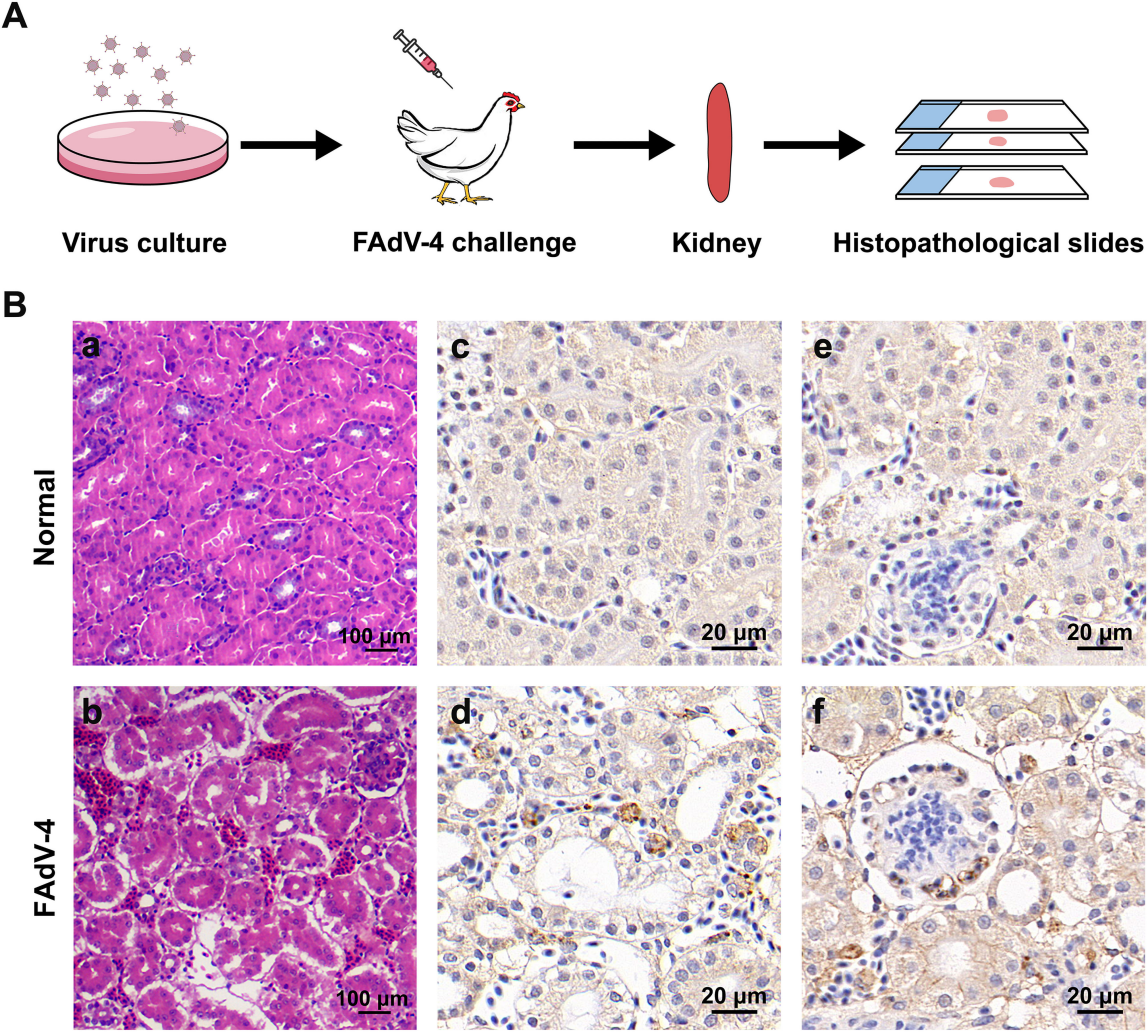
Histopathological changes to the FAdV4-infected kidney. (A) Representative histopathological images post-FAdV-4 infection. (B) Histopathological changes of the FAdV4-infected kidney. (a and b) Hematoxylin-eosin staining. (a) Normal kidney. (b) The kidney post-FAdV-4 infection (scale bar, 100 µm). (c–f) Immunohistochemical assay results, where brownish-yellow staining indicates positive signals. (c) Normal tubules. (e) Kidney abnormalities post-infection. (d) Normal glomeruli. (f) The glomeruli were dilated and denatured, with enlarged lacunae in the kidney sacs post-infection. Scale bar, 20 µm.

Histopathological changes in the kidneys of chickens following infection were analyzed by staining with hematoxylin and eosin and immunohistochemical analysis (Fig. 3). The structures of the tubular epithelial cells and capillaries in normal kidneys were intact, with no exudate observed in the tubule lumens. However, at 24 hpi, the tubular epithelial cells exhibited denaturation, necrosis, and swelling. The kidney tubular lumens displayed a significant presence of red blood cells and shedding of epithelial cells. Circular vacuoles appeared in the cytoplasm of kidney epithelial cells, and some nuclei were displaced or showed nucleolysis. Additionally, the glomeruli appeared dilated and denatured, with enlarged lacunae in the kidney sacs. Immunohistochemical analysis revealed brownish-yellow staining of the kidney interstitium, epithelial cells, and some glomerular cells post-FAdV-4 infection, indicating that FAdV-4 primarily targeted these cells in the kidney. The structural damage and scar formation indicated potential kidney dysfunction.

### mRNA levels of cytokines in the kidney post-infection

**Fig 4:**
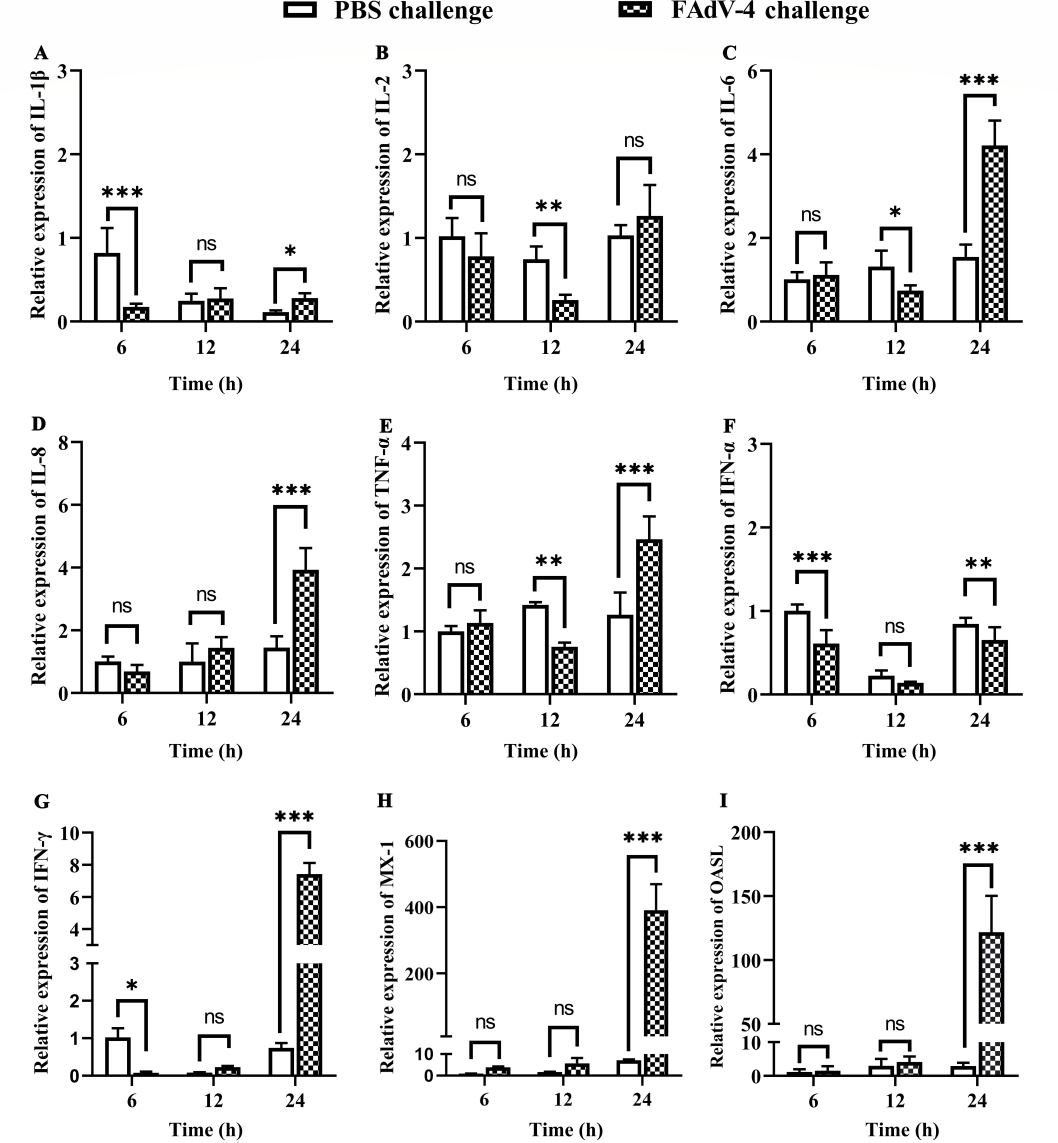
Changes to kidney cytokine levels post-FAdV-4 infection. (A) IL-1β, (B) IL-2, (C) IL-6, (D) IL-8, (E) TNF-α, (F) IFN-α, (G) IFN-γ, (H) MX-1, and (I) OASL. The data were analyzed with the two-way ANOVA using GraphPad Prism 8.0.2 software. The data are presented as the means ± SD of three independent experiments. ns, *P* > 0.05; *P < 0.05; **P < 0.01; and ***P < 0.001.

The mRNA levels of cytokines in the kidney were analyzed at 6, 12, and 24 hpi, as depicted in Fig. 4. The mRNA levels of the inflammatory factors IL-1β and IL-2 were relatively low in the kidney, whereas mRNA levels of IL-6, IL-8, and TNF-α were significantly upregulated at 24 hpi by 2.7-, 2.7-, and 2.0-fold, respectively. The mRNA expression levels of IFN-α at 6, 12, and 24 hpi were significantly downregulated during early viral infection. IFN-γ expression level was downregulated at 6 hpi and then significantly upregulated at 24 hpi by 10.0-fold. Additionally, MX-1 and OASL mRNA expression levels were significantly upregulated at 24 hpi by 54.0- and 41.7-fold, respectively. These findings indicated that FAdV-4 at 24 hpi elicited significant upregulation of the inflammatory cytokines IL-6, IL-8, and TNF-α, as well as the antiviral cytokines IFN-γ, MX-1, and OASL. Notably, upregulation of the IFN-stimulated cytokines MX-1 and OASL elicited a robust antiviral innate immune response.

### FAdV-4 infection promoted cellular autophagy in the kidney

**Fig 5:**
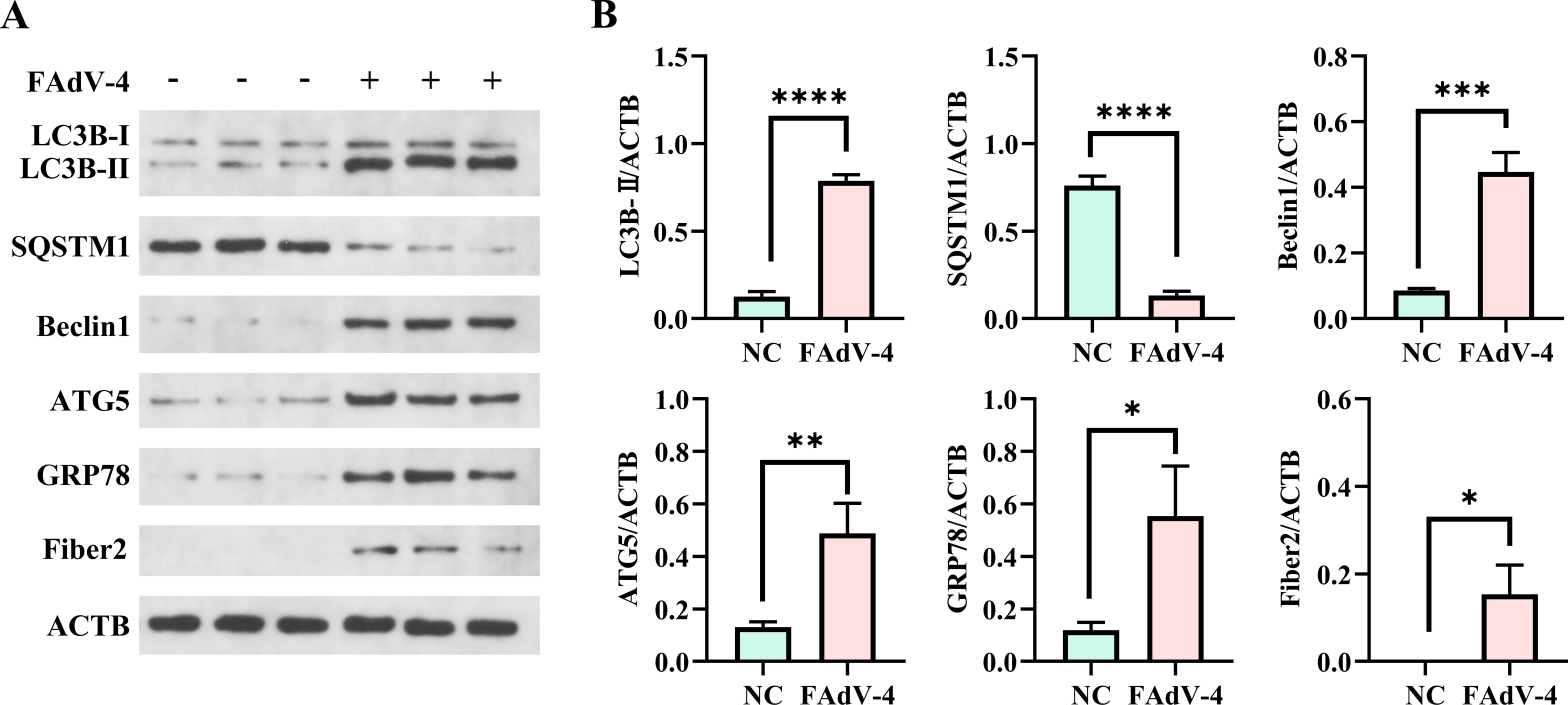
FAdV-4 promoted cellular autophagy in chicken kidney cells. (A) The expression levels of the autophagy-related proteins of LC3B, SQSTM1, Beclin1, ATG5, and GRP78 in the spleen post-infection were measured by western blotting analysis. (B) The protein bands were quantified against ACTB expression using ImageJ software. Data are means ± SD of the results of three independent experiments. ns, no significant difference; *P* > 0.05; *P < 0.05; **P < 0.01; and ***P < 0.001.

To investigate the impact of FAdV-4 infection on cellular autophagy in the kidney cells of SPF chickens, western blotting analysis was utilized to analyze changes in the expression levels of the autophagy-related proteins LC3B, SQSTM1, Beclin1, and ATG5, as well as the ER stress marker GRP78 (Fig. 5). The expression of LC3-II, a key marker of autophagy, was upregulated in FAdV-4-infected kidney tissues by 2.7-fold. Furthermore, the expression levels of ATG5 and BECN1 were upregulated by 3.1- and 3.3-fold, respectively, while the expression of SQSTM1 was decreased by 2.6-fold as compared to uninfected tissues, indicating induction of complete autophagy post-FAdV-4 infection in the kidney. Interestingly, GRP78 expression was increased by 2.3-fold following FAdV-4 infection, suggesting a potential link between ER stress-mediated autophagy and viral replication *in vivo*.

### Impact of cellular autophagy regulators on FAdV-4 replication

The CCK-8 assay was utilized to assess the impact of cellular autophagy regulators rapamycin and 3-MA on the viability of primary chick kidney cells (Fig. S1). As the concentration of the autophagy regulators was increased, cell viability gradually decreased. Specifically, when the concentration of rapamycin ranged from 5 to 25 nM, cell viability remained greater than 90%. However, at a concentration of 50 nM, cell viability decreased to 86.03%. Similarly, at 3-MA concentrations of 1 and 2 mM, cell viabilities were 98.82% and 95.49%, respectively. With a further increase in 3-MA concentrations to 3 mM, cell viability decreased to 85.67%. Overall, the CCK-8 assay indicated that the optimal concentrations of rapamycin and 3-MA were 10 nM and 1 mM, respectively, which might exhibit minimal impact on primary kidney cell viability.

**Fig 6:**
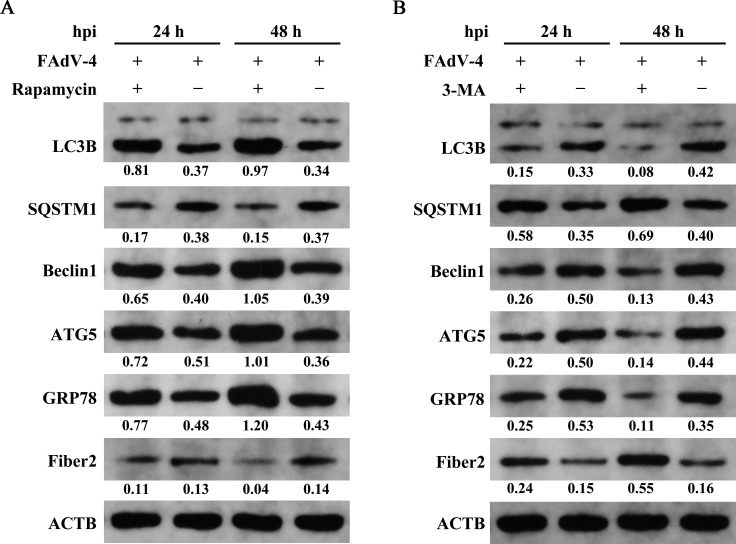
Impact of autophagy regulators on FAdV-4 replication. (A) The expression levels of autophagy-related proteins (LC3B, ATG5, BECN1, and SQSTM1), GRP78, and viral Fiber2 after rapamycin treatment as detected by western blotting analysis. (B) The expression levels of autophagy-related proteins (LC3B, ATG5, BECN1, and SQSTM1), GRP78, and viral Fiber2 after 3-MA treatment as detected by western blotting analysis. The abundance of each protein band was quantified with the ImageJ software and normalized to ACTB; the protein/ACTB ratio was shown below each band.

To investigate the impact of cellular autophagy on FAdV-4 proliferation, primary chick kidney cells were cultured with rapamycin (10 nM) or 3-MA (1 mM) to assess the expression levels of the autophagy-related proteins LC3B, SQSTM1, Beclin1, ATG5, GPR78, and viral Fiber2 (Fig. 6). The results showed that Fiber2 protein expression increased with enhanced autophagy post-FAdV-4 infection without the addition of autophagy regulators. In the rapamycin group, Fiber2 protein expression was elevated but inhibited in the 3-MA group. The rapamycin-treated cells exhibited upregulation of LC3B, Beclin1, ATG5, and GPR78 by 2.20-, 1.63-, 1.38-, and 1.60-fold, respectively, with a 2.24-fold decrease in SQSTM1 at 24 hpi. Moreover, the protein expression levels of LC3B, Beclin1, ATG5, and GPR78 were upregulated by 2.85-, 2.69-, 2.81-, and 2.79-fold, respectively, with a 2.47-fold decrease in SQSTM1 at 48 hpi. Conversely, the protein expression levels of LC3B, Beclin1, ATG5, and GPR78 were downregulated by 2.20-, 1.92-, 2.27-, and 2.12-fold, respectively, while SQSTM1 expression was increased by 1.66-fold in the 3-MA group as compared to the control group at 24 hpi. Meanwhile, the expression levels of LC3B, Beclin1, ATG5, and GPR78 were decreased by 5.25-, 3.31-, 3.14-, and 3.18-fold, respectively, with a 1.73-fold increase in SQSTM1 at 48 hpi.

### Virus replication and cellular autophagy in infected chickens

**Fig 7:**
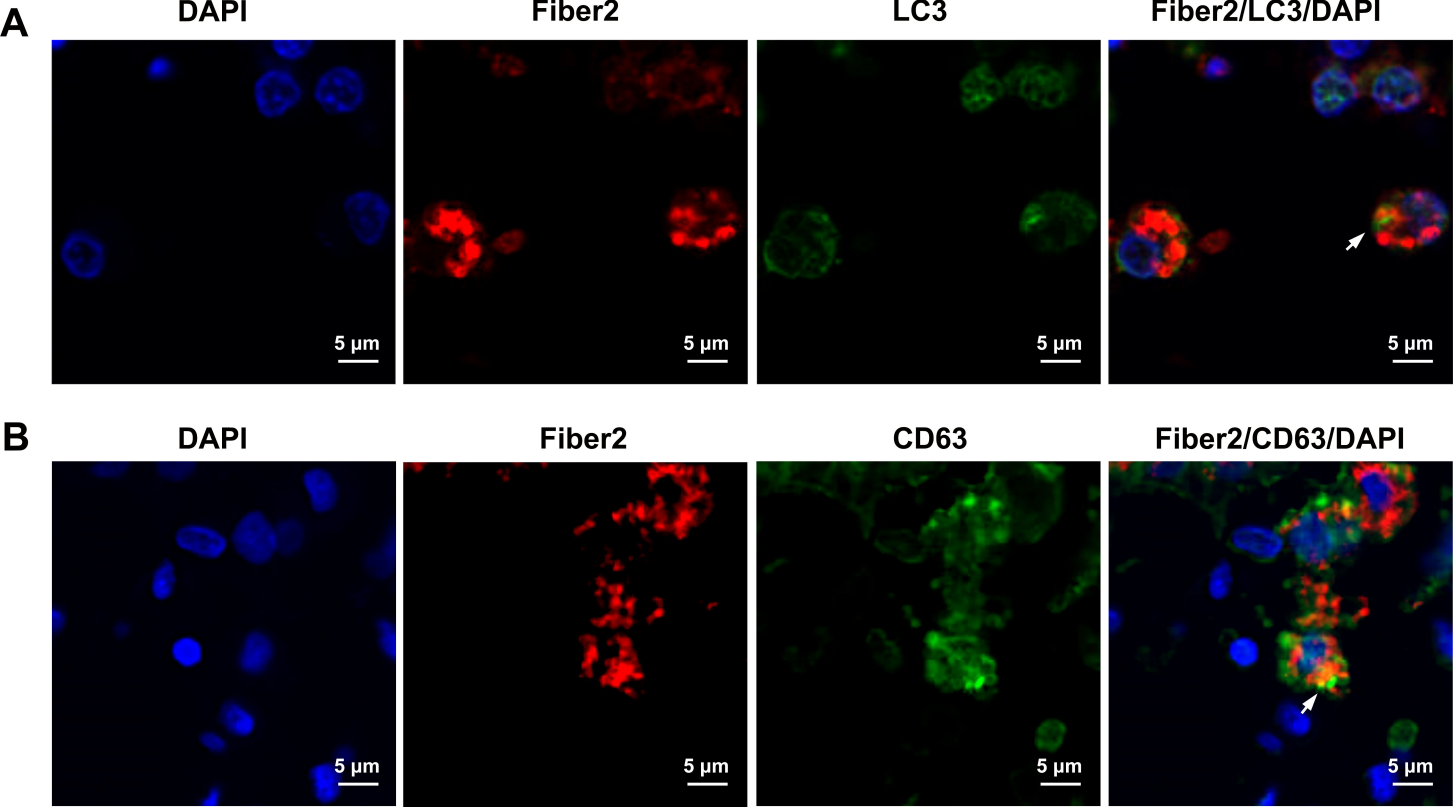
Immunofluorescence confocal images of co-localization in primary kidney cells post-FAdV-4 infection. (A) Fiber2 was colocalized with LC3B post-infection. (B) Fiber2 was colocalized with CD63 post-infection. Scale bar, 5 µm. Arrows point to cellular co-location (orange fluorescence signals). Images show the results of one out of three replicates.

To investigate the correlation between autophagy and viral replication, specific antibodies were used to identify the subcellular localization of the viral proteins Fiber2 and LC3B in kidney tissue cells infected with FAdV-4 (Fig. 7). Fluorescent signals indicating aggregation of LC3B, a marker of cellular autophagy formation, were observed in FAdV-4-infected kidney cells. Both LC3B and Fiber2 proteins were co-localized in kidney cells, suggesting that FAdV-4-induced cellular autophagy could enhance viral replication in kidney cells. Additionally, the distribution of CD63, an exosome-specific marker protein involved in vesicular trafficking and cargo sorting, was examined post-FAdV-4 infection. The co-localization of CD63 and Fiber2 in FAdV-4-infected kidney cells implies a potential role of autophagosome-like vesicles in the replication of FAdV-4.

## DISCUSSION

Autophagy is a dynamic cellular process for the degradation of damaged or abnormal proteins and senescent organelles in the cytoplasm through autophagy-lysosome pathways. Damaged organelles and cell debris are primarily degraded by lysosomes facilitated by the formation of single-membrane or double-membrane vesicles (29). The formation of autophagosomes helps maintain cell homeostasis in response to stimuli, such as pathogen infection (30). Autophagy serves as a natural defense mechanism against viruses and is integral to the innate immune response. Certain viruses have developed strategies to manipulate cellular autophagy via specific viral proteins to benefit replication (31, 32). Autophagy plays a significant role in the response of host cells to FAdV-4 infection, as evidenced by *in vitro* studies of LMH cells (12, 23). The low basal level of autophagy in normal kidney cells indicates the importance of basal autophagy in the maintenance of homeostasis of host cells. Upon the FAdV-4 challenge, the autophagy-related marker proteins LC3B, ATG5, and BECN1 were upregulated in kidney tissues, accompanied by the degradation of p62/SQSTM1. LC3B and ATG5 are crucial for the formation of autophagosomes and regulation of autophagy pathways, while LC3B is also involved in membrane expansion and closure of vesicles. ATG5 promotes vesicle formation and maturation, and BECN1 regulates the early stages of autophagy and is also associated with the formation of vesicles. Overall, these proteins are closely linked to autophagosome formation (33). The results of the present study indicated that FAdV-4 infection boosts autophagic flux, leading to a complete autophagic response in host kidney cells. Post-FAdV-4 infection, cellular autophagy is enhanced, resulting in increased expression levels of Fiber2, which indicates the promotion of viral replication. In this study, rapamycin promptly activated cellular autophagy and decreased Fiber2 levels during FAdV-4 infection, while 3-MA, an autophagy inhibitor, exhibited an opposite effect, inferring that host cellular autophagy might be excessively active during virus infection and degrade Fiber2, a crucial immune component of FAdV-4 (19). Previous research has shown that the autophagy cargo receptor p62/SQSTM1 is involved in the degradation of the *Avibirnavirus* capsid protein VP2. The interaction between SQSTM1 and VP2 enhances autophagy induction, thereby facilitating degradation of the viral protein VP2 (34). Coincidentally, the host factor OASL can directly target the ubiquitinated VP2 protein of infectious bursal disease virus for degradation through the OASL-p62/SQSTM1 autophagy pathway, thereby limiting viral replication (35). The host cellular autophagy acts like a “double-edged sword” in the process of viral infection (19). However, further investigations are needed to elucidate the specific interactions among Fiber2, LC3B, and SQSTM1 in the regulation of cellular autophagy post-FAdV-4 infection, as well as the underlying mechanism.

To further elucidate the relationship between cellular autophagy and viral infection in kidney cells post-FAdV-4 infection, co-localization analysis was conducted using confocal immunofluorescence microscopy to examine the interaction of the viral Fiber2 protein with the autophagy marker LC3 and the exosome marker CD63. The results revealed that Fiber2, a structural protein crucial for FAdV-4 early infection and replication, co-localized with LC3B or CD63. LC3B, an essential component of the mature autophagosome membrane, was identified as a marker of autophagy (36). CD63, a classic exosome marker, is reportedly involved in the formation and trafficking of vesicles and exocytosis (37). The results of the present study suggested that FAdV-4 infection triggers cellular autophagy by host cells, with autophagosome-like vesicles potentially playing a role in viral replication. This implies that the autophagy pathway is necessary to facilitate replication and enhance infection efficiency during FAdV-4 infection, particularly in the early stages. While these findings emphasize the significance of the autophagy pathway in FAdV-4 infection, the precise mechanism linking cellular autophagy to viral replication needs to be examined in depth.

**Fig 8:**
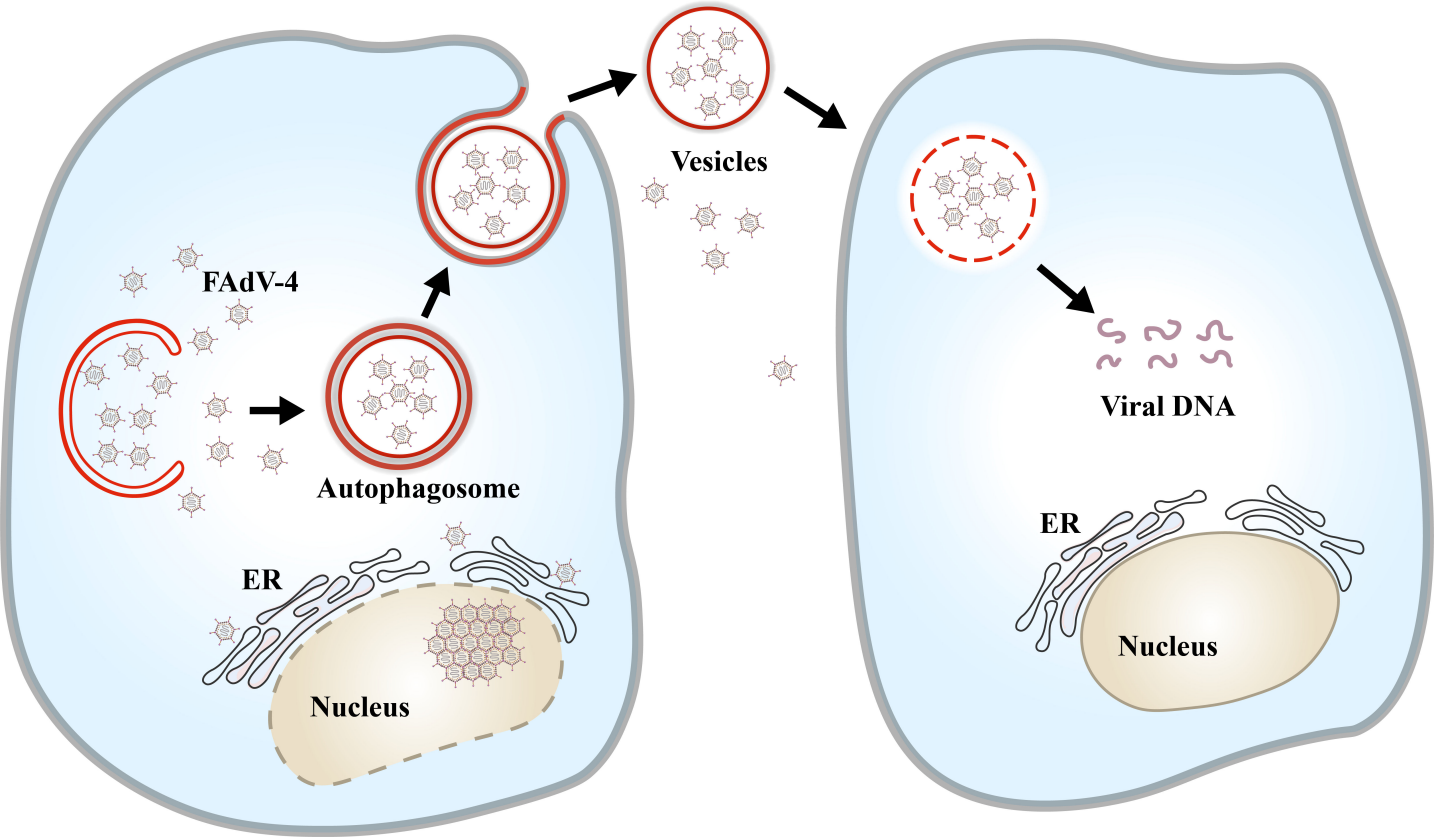
Schematic diagram of the vesicle-mediated kidney cell-to-cell transmission mode of FAdV-4.

Vesicles secreted by host cells play a protective role in virus transmission and transport. Recent studies have demonstrated that vesicles can sequester virions from the intercellular environment and facilitate the transfer of signaling molecules between cells and potentially modify the functions of target cells (38). The aggregation and packaging of virus particles within cytoplasmic vesicles enable the transportation of multiple viruses to new host cells. This study presented a novel finding of vesicle-dependent transmission of FAdV-4, which might enhance virus replication, thereby further clarifying the pathogenesis and tissue tropism of FAdV-4. Autophagosome-like vesicles were observed in the intracellular and extracellular primary kidney cells post-FAdV-4 infection. Some clusters of mature infectious virus particles were observed within these vesicles, suggesting that these vesicles play a role in transporting viruses through cell-to-cell transmission and might facilitate subsequent infection (39, 40). A previous study also observed autophagosome-like vesicles in the cytoplasm of LMH cells. Interestingly, we observed the autophagosome-like vesicles delivering virus particles in both the intracellular and extracellular kidney primary cells. There might be a novel mechanism of vesicle-mediated intercellular transmission of virulent FAdV-4 that hijacks autophagosomal vesicles (Fig. 8). Vesicle transport is a novel mode of cellular communication that allows cells to influence neighboring and distant cells, consequently increasing infectivity (41). A prior study demonstrated that vesicle-like autophagosomes bypassed fusion with lysosomes and instead fused with the plasma membrane to release vesicle-carrying poliovirus particles (39). Utilizing intercellular contact or vesicle-dependent transmission can enhance the transmission efficiency of viruses while also allowing evasion of neutralization by antibodies, thus facilitating effective immune escape (42). Previous study has shown that FAdV-4 infection leads to a reduction in PKR protein levels in host cells to evade innate immune responses (43).

Interestingly, FAdV-4 particles were also observed in the abnormal mitochondria of kidney cells during infection, indicating that FAdV-4 may manipulate host cell mitochondria and potentially induce injury. This study is the first to report virus particles within mitochondria at the ultrastructural level. Infiltration of mitochondria in kidney tubular epithelial cells by FAdV-4 could disrupt homeostasis, leading to structural and functional impairments of host cells, cell death, kidney tissue damage, and potentially organ failure.

Taken together, our findings primarily demonstrate that FAdV-4 infection induces damage to kidney tissues, characterized by epithelial cell degeneration and necrosis, glomerular swelling and degeneration, and ER stress, while also triggering a robust inflammatory response in kidney cells. Infection by hypervirulent FAdV-4 leads to damage and disintegration of the nuclear membrane, release of nucleic acids into the cytoplasm and bloodstream, and elicitation of an inflammatory response (44). Meanwhile, the inflammatory factors IL1-β, IL-2, IL-6, and TNF-α were upregulated in the kidney post-FAdV-4 infection. These cytokines are crucial signaling molecules in immune regulation and the inflammatory response. Besides, the expression levels of the autophagy-related markers LC3B, ATG5, and BECN1 were increased, while SQSTM1 was degraded, suggesting that changes to the autophagosome levels of immune cells, mediated by cytokines, may play a pivotal role in immunity and inflammation. However, the relationship between the inflammatory response triggered by FAdV-4 infection and the autophagy signaling pathway remains unclear. Previous research has shown that certain viruses can utilize autophagy to combat innate immunity (45), and there exists a complex interplay between autophagy and various pattern recognition receptors, involving both cooperative and antagonistic interactions. Our study offers novel insights into the pathogenesis of hypervirulent FAdV-4 from the perspective of kidney injury post-infection.

## SUPPLEMENTAL MATERIAL

Fig. S1 spectrum.00058-25-s0001.tif Effects of rapamycin and 3-MA on kidney cell viability as determined with the CCK-8 assay.

Supplemental material spectrum.00058-25-s0002.docx Fig. S1 legend.

ASM does not own the copyrights to Supplemental Material that may be linked to, or accessed through, an article. The authors have granted ASM a non-exclusive, world-wide license to publish the Supplemental Material files. Please contact the corresponding author directly for reuse.
