# Supplementary figures and images for: Mechanistic insights into the kidney injury in chickens induced by hypervirulent fowl adenovirus serotype 4
Source: Microbiol Spectr. 2025 Mar 25;13(5):e00058-25. doi: 10.1128/spectrum.00058-25 (PMC12054176; doi:10.1128/spectrum.00058-25)

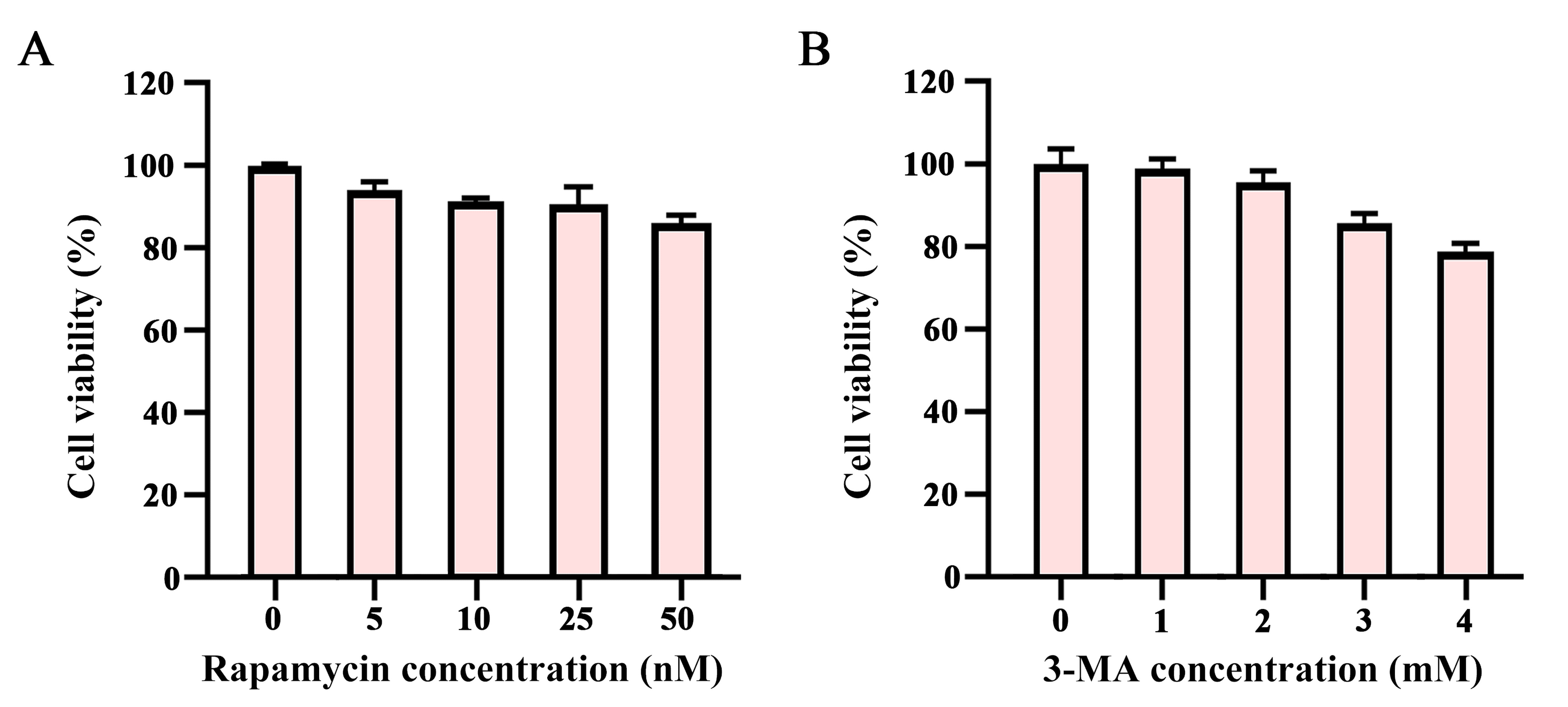

Supplement: Fig. S1 — Effects of rapamycin and 3-MA on kidney cell viability as determined with the CCK-8 assay. [file spectrum.00058-25-s0001.tif]
