# Supplementary material for: Mechanistic insights into the kidney injury in chickens induced by hypervirulent fowl adenovirus serotype 4
Source: Microbiol Spectr. 2025 Mar 25;13(5):e00058-25. doi: 10.1128/spectrum.00058-25 (PMC12054176; doi:10.1128/spectrum.00058-25)
Supplement: Supplemental material — Fig. S1 legend. [file spectrum.00058-25-s0002.docx]

**Figure S1. Effects of rapamycin and 3-MA on kidney cell viability as determined with the CCK-8 assay.** (A) Rapamycin. (B) 3-MA. The data are presented the means ± SD of five independent biological experiments. Cell viability (%) = [(OD_Sample_ − OD_Blank_) ÷ (OD_Normal_ − OD_Blank_)] × 100%.
